# The Cholera in Naples

**Published:** 1838-04

**Authors:** 


### THE CHOLERA IN NAPLES.

[In our preceding Number, (p. 260,) we have given a brief account of the prevalence of cholera at Naples, from official documents; for the following additional particulars we are indebted to the kindness of a friend, resident in that city.]

*Extract of a Letter from a Physician, dated Naples, 17th November, 1837.*

"The first well-attested case of cholera occurred on the 2d October, 1836, on the person of a *Doganiere*, or custom-house officer. Here, as in every other place, the faculty was divided into contagionists and non-contagionists. Dr. de Renzi, the editor of the *Filiatre Sebezio*, was then a very violent contagionist, and attempted to prove that the disease was introduced into Naples by a person who came from Puglia. I took considerable pains to ascertain the facts connected with this case. I went to the house where the man lodged who came from Puglia. I found that he had been ill and was sent to a hospital, where I went; and, after a great deal of trouble, I got hold of the books of the hospital, which gave an account of his admission, the medicines he had taken, and the day of his dismissal. The keeper of the hospital flatly denied its being a case of cholera, and gave me the name of the physician who had attended him. I went to him, and had some conversation with him on the subject: he was decidedly of opinion that it was not cholera which this man had, but a common biliary fever; he was treated, in the usual way, with diaphoretics, purgatives, and afterwards with infusion of gentian. I must, however, in justice to De Renzi, say, that, in the same house where this man lodged, another man was taken ill with symptoms strongly resembling those of Asiatic cholera, and died on the second or third day of his complaint. The government, however, did not acknowledge this to have been cholera, although the medical man who attended him, and to whom I wrote for his opinion of the case, said that he believed the disease to have been so. The custom-house officer, however, was not attacked till nearly a fortnight after this last case; and no other person (several slept in the same room with these men,) was attacked in this house.

"Since that time De Renzi has recanted, and has published his recantation in a late number of the *Filiatre;* and by far the greater number of the Neapolitan physicians are now persuaded that the disease is not contagious. This change of opinion among the medical men has already produced its effect on the government; and they are now, apparently, as anxious to remove their quarantines and cordons sanitaires as they formerly were to lay them on. For my own part, I am in some doubts respecting the nature of the disease. I have seen it in four different places, viz. in Bengal in 1825, in China in 1826, in Naples in 1836, and lately in Sorrento during the past summer; and although I have generally declared myself a non-contagionist, yet several cases which I lately witnessed have rather tended to shake this opinion. Be that as it may, one thing is certain, that, whether the disease be contagious or not, we have never been able, with all our cordons and quarantines, to stop its progress; and, therefore, the sooner all restrictions of this kind are removed the better it will be for the world. The disease, as I said, began here on the 2d of October, 1836, and raged with great violence till the beginning of December, when it began to decline; and by the end of the year it had almost disappeared. The rest of the winter was passed quietly; but, on the 13th of April last, it again shewed itself; and by the end of May it was occasioning great apprehensions; in June and July it was more severe than it had ever been before; but in August it again began to subside; and we are now, thank God, entirely free from it. During the first attack of the complaint the mortality was supposed to have amounted to about eight thousand, and in the second attack to nearly twice that number. Many estimate the mortality much higher; but, as the government evidently concealed a number of the deaths, it is impossible to say exactly how many were carried off. The mortality in proportion to the cases is supposed to have been nearly as six to ten. This does not say much for our improvement in the treatment of the disease; indeed, I am not sure that we have advanced one step; for, even with all the disadvantages of climate against them, I believe that the medical men in India were as successful in their treatment (from the very commencement, too,) as their European brethren have since been. The mortality at Palermo was dreadful: out of a population of 150,000, about 30,000 of whom are supposed to have left the town at the breaking out of the complaint, the deaths were nearly 25,000, or about a fifth part of those who remained!"

